# Seasonal Variation in PM_2.5_ Composition Modulates Oxidative Stress and Neutrophilic Inflammation with Involvement of TLR4 Signaling

**DOI:** 10.3390/antiox15010089

**Published:** 2026-01-09

**Authors:** Duo Wang, Zirui Zeng, Aya Nawata, Ryoko Baba, Ryuji Okazaki, Tomoaki Okuda, Yasuhiro Yoshida

**Affiliations:** 1 Department of Radiobiology and Hygiene Management, Institute of Industrial Ecological Sciences, University of Occupational and Environmental Health, Japan, 1-1 Iseigaoka, Yahatanishi-ku, Kitakyushu 807-8555, Japanryuji-o@med.uoeh-u.ac.jp (R.O.); 2 The First Department of Internal Medicine, School of Medicine, University of Occupational and Environmental Health, Japan, 1-1 Iseigaoka, Yahatanishi-ku, Kitakyushu 807-8555, Japan; 3 Department of Pathology and Oncology, School of Medicine, University of Occupational and Environmental Health, Japan, 1-1 Iseigaoka, Yahatanishi-ku, Kitakyushu 807-8555, Japan; aya.y0116@gmail.com; 4 Department of Anatomy, School of Medicine, University of Occupational and Environmental Health, Japan, 1-1 Iseigaoka, Yahatanishi-ku, Kitakyushu 807-8555, Japan; 5 Department of Applied Chemistry, Faculty of Science and Technology, Keio University, 3-14-1 Hiyoshi, Kohoku-ku, Yokohama 223-8522, Japan; 6 Department of Immunology and Parasitology, School of Medicine, University of Occupational and Environmental Health, Japan, 1-1 Iseigaoka, Yahatanishi-ku, Kitakyushu 807-8555, Japan

**Keywords:** reactive oxygen species (ROS), particulate matter 2.5 (PM_2.5_), lung inflammation, seasonality, air pollution toxicity, innate immune response

## Abstract

Seasonal fluctuations in the chemical composition of fine particulate matter (PM_2.5_) are known to influence its toxicological properties; however, their integrated biological effects remain incompletely understood. In this study, PM_2.5_ was continuously collected over two consecutive years at a single urban site in Japan and classified by season. The samples were comprehensively characterized for ionic species, metals, carbonaceous fractions, and polycyclic aromatic hydrocarbons (PAHs), and their pulmonary effects were evaluated in vivo following intratracheal administration in mice. Seasonal PM_2.5_ exhibited pronounced compositional differences, with higher levels of secondary inorganic aerosol components in summer and enrichment of PAHs and mineral-associated components in winter. These seasonal differences translated into distinct biological responses. Reactive oxygen species (ROS) production (1.6–2.7-fold increase) and bronchoalveolar lavage (BAL) neutrophil infiltration were strongly associated with PAH-rich PM_2.5_, whereas interleukin-1α (IL-1α) showed robust positive correlations with mineral components, including K^+^, Ca^2+^, and Mg^2+^, which were predominantly enriched in winter PM_2.5_. In contrast, secondary inorganic aerosol species displayed a limited capacity to induce IL-1α. Compared with summer samples, winter PM_2.5_ induced significantly higher levels of ROS production and IL-1α (approximately 1.5–2.6-fold increase). Using TLR2- and TLR4-deficient mice, we further demonstrated that PM_2.5_-induced increases in BAL cell counts, ROS, IL-6, and TNF-α were partially attenuated in TLR4 knockout mice, indicating a contributory but not exclusive role for TLR4 signaling in PM_2.5_-driven pulmonary inflammation. Collectively, these findings demonstrate that seasonal variations in PM_2.5_ composition, not particle mass alone, critically shape oxidative stress and innate immune responses in the lungs. In particular, winter PM_2.5_ enriched in mineral-associated components preferentially activates IL-1α-mediated alarmin pathways, underscoring the importance of the particle composition in determining seasonal air pollution toxicity.

## 1. Introduction

Exposure to particulate matter (PM) poses serious risks to human health [1]. Major PM sources include vehicle emissions, industrial activities, construction operations, and natural events such as wildfires and volcanic eruptions [2,3]. PM varies widely in size and composition, and it is typically classified according to aerodynamic diameter. Among these, particles smaller than 2.5 µm has been extensively linked to adverse respiratory and systemic health effects [4,5,6]. Because of their small size, PM_2.5_ can penetrate deep into the respiratory tract and reach the alveoli, where they contribute to infections, airway inflammation, and chronic lung diseases [7,8,9].

The physicochemical characteristics of PM_2.5_ differ substantially depending on sampling location [10,11], season [12], and year [13]. Therefore, understanding PM_2.5_ toxicity requires direct experimental evaluation. Several studies have compared seasonal PM_2.5_ effects. Melzi et al. showed that summer and winter PM_2.5_ differ in their capacity to induce oxidative stress, inflammation, and DNA damage in cell lines [14]. Farina et al. reported that heme oxygenase-1 (HO-1), a key antioxidant and stress-response enzyme, was markedly increased following exposure to aerosolized summer PM in mice [15]. Marchetti et al. used PM_2.5_ collected over four seasons and demonstrated that biological responses correlated positively with PAHs and metals characteristic of combustion-derived pollution [16]. The use of PM_2.5_ samples collected over two consecutive years enhances the robustness of seasonal analyses by reducing the influence of interannual variability in meteorological conditions and emission sources, thereby enabling identification of consistent and reproducible seasonal patterns in particle composition and toxicity [17]. However, no study has continuously collected PM_2.5_ over two full years and examined its detailed biological effects in animal models.

Various studies have also examined metal components of PM_2.5_, including Pb, Cu, Cd, and Ni [18]. For example, PM_2.5_ analysis in Puerto Rico revealed higher levels of Ni and V in industrial areas, implicating metal pollution in local respiratory symptoms [19]. However, there remains a lack of integrated analyses linking year-round PM_2.5_ composition with biological responses—particularly oxidative stress—and their interrelationships.

Neutrophils and macrophages express Toll-like receptors (TLRs) that recognize pathogen-associated molecular patterns such as lipopolysaccharide (LPS). TLR2 recognizes lipoproteins and glycolipids, while TLR4 triggers intracellular signaling upon LPS binding, leading to pro-inflammatory cytokine production [20]. We previously showed that murine neutrophils endocytose PM and that this process is diminished in TLR4-deficient mice [21]. We also reported that LPS attached to PM_10_ suppresses splenocyte immune responses [22] and that LPS levels bound to PM_2.5_ strongly influence PM-induced immunosuppression [23]. These findings indicate that the quantity and nature of PM-adsorbed constituents critically shape immune responses.

PM_2.5_ exposure is widely known to induce reactive oxygen species (ROS) [24,25,26,27,28]. Once deposited in the lungs, PM_2.5_ generates ROS directly through redox-active components and indirectly through inflammatory cell activation, overwhelming antioxidant defenses and causing lipid, protein, and DNA damage [29]. ROS serves as a key upstream signal that activates pathways leading to cytokine production, including IL-6 and TNF-α. Our laboratory previously demonstrated that neutrophil endocytosis of 1 µm PM stimulates IL-6 and TNF-α release [21].

In this study, we collected PM_2.5_ continuously over two years from a single site in Japan and categorized samples into four seasons per year. We analyzed PM_2.5_ composition and evaluated biological effects following intratracheal instillation in mice. We further examined correlations between seasonal PM_2.5_ characteristics and ROS production.

## 2. Materials and Methods

### 2.1. Sampling and Preparation of PM_2.5_

The sampling site was located in an urban residential area approximately 15 km southwest of central Tokyo [30]. Aerosol samples were collected by large cyclones (flow rates 1200 L/min) on the rooftop of a 22 m building at Keio University between February 2021 and February 2023, following previously described protocols [31,32]. PM_2.5_ samples were collected for a total 10 samples, among which PM_2.5_ samples collected twice between February and April 2021 were combined as PM-A1 to correspond to samples collected during the same period in 2022. All samples were suspended in PBS at 10 mg/mL.

### 2.2. Analysis of PM_2.5_ Content

Collected particles were characterized using ion chromatography (Cl^−^, NO_3_^−^, SO_4_^2−^, Na^+^, NH_4_^+^, K^+^, Mg^2+^, Ca^2+^), thermal–optical analysis for OC1–4 and EC1–3, high-performance liquid chromatography (HPLC) for polycyclic aromatic hydrocarbons (PAHs, including acenaphthene, fluorene, phenanthrene, anthracene, fluoranthene, pyrene, benz[a]anthracene, chrysene, benzo[b]fluoranthene, benzo[k]fluoranthene, benzo[a]pyrene, dibenz[a,h]anthracene, benzo[g,h,i]perylene, indeno[1,2,3-cd]pyrene), and ICP-MS for metals (Al, Si, Ti, V, Cr, Mn, Fe, Ni, Cu, Zn, Pb), following established methodologies [32,33]. Endotoxin levels were quantified using the manufacturer’s protocol (Associates of Cape Cod).

#### Quality Assurance and Quality Control (QA/QC)

Quality assurance and quality control (QA/QC) procedures were implemented for all chemical analyses of PM_2.5_ samples. Water-soluble ions (Cl^−^, NO_3_^−^, SO_4_^2−^, Na^+^, NH_4_^+^, K^+^, Mg^2+^, Ca^2+^) were quantified by ion chromatography, and analytical accuracy was verified using certified reference material CRM#28 (Urban Aerosols, NIES, Tsukuba, Japan). Limits of detection (LOD) for each ion species were determined to be five times the standard deviation of replicate analyses of standard solutions, and the coefficients of variation for repeated measurements were below 7%.

Elemental concentrations were determined by inductively coupled plasma–mass spectrometry (ICP-MS) following microwave acid digestion. Quantification was performed using multi-point calibration curves, and analytical accuracy was assessed using certified reference materials, yielding recovery rates between 87% and 97%. Instrument calibration and blank corrections were routinely performed [32].

Carbonaceous fractions (OC and EC) were analyzed using a thermal–optical method following the IMPROVE protocol. PAHs were measured by HPLC. Endotoxin and β-glucan contents were determined using commercial assay kits according to the manufacturers’ instructions. Detailed analytical procedures have been described previously [31].

### 2.3. Mice and Intratracheal Administration of PM_2.5_

BALB/c, TLR2 KO, and TLR4 KO mice (male, 7–11 weeks old) were obtained from Japan SLC (Hamamatsu, Japan). PM_2.5_ was suspended at 1 mg/mL in PBS and administered intratracheally (100 μg/100 μL/mouse) under 5% sevoflurane anesthesia. BALF was collected 24 h post-administration.

### 2.4. Measurement of BAL Cell Number and ROS Production

BALF was collected by cannulating the trachea and gently instilling sterile PBS (1.0 mL per instillation) into the lungs. After centrifuging, BALF was separated into fluids and BAL cells. The fluids were applied for subsequent analysis. BAL cells were pelleted, resuspended, and counted. ROS production was assessed using a DCFH-DA assay (Dojindo, Kumamoto, Japan), with fluorescence measured on a plate reader following established protocols [34].

### 2.5. Flow Cytometry

BAL cells were stained with PE-anti-F4/80 and VioletFluor450-anti-Gr-1 (San Diego, CA, USA) at 4 °C for 30 min, washed, and analyzed using a CytoFLEX cytometer. Gating strategy was shown as Supplementary Figure S1.

### 2.6. Enzyme-Linked Immunosorbent Assay (ELISA)

BALF cytokines (IL-6, TNF-α, IL-1α, IL-12) were quantified using commercial ELISA kits (BioLegend, San Diego, CA, USA), following the manufacturer’s instructions.

### 2.7. Pathology Analysis

Lungs were fixed, paraffin-embedded, sectioned at 5 μm, and stained with hematoxylin and eosin. Ten non-overlapping 200× fields were scored 0–4 for inflammation severity according to published criteria [35,36,37].

### 2.8. Statistics

Results are expressed as mean ± SD. Correlation analyses were performed to evaluate the relationships between PM_2.5_ chemical components and biological endpoints, including BAL cellular responses, cytokine levels (IL-6, TNF-α, IL-1α, and IL-12), and ROS production. Pearson correlation was employed due to the study’s focus on linear associations, while noting that potential non-linear relationships cannot be excluded and warrant further investigation in future studies. Statistical significance was assessed by one-way ANOVA with Fisher’s LSD test, with *p* < 0.05 considered significant.

## 3. Results

### 3.1. PM_2.5_ Collected During the Summer (PM-C) Showed Elevated Sulfur (S) and Reduced Calcium (Ca) and OCP Levels

**Figure 1:**
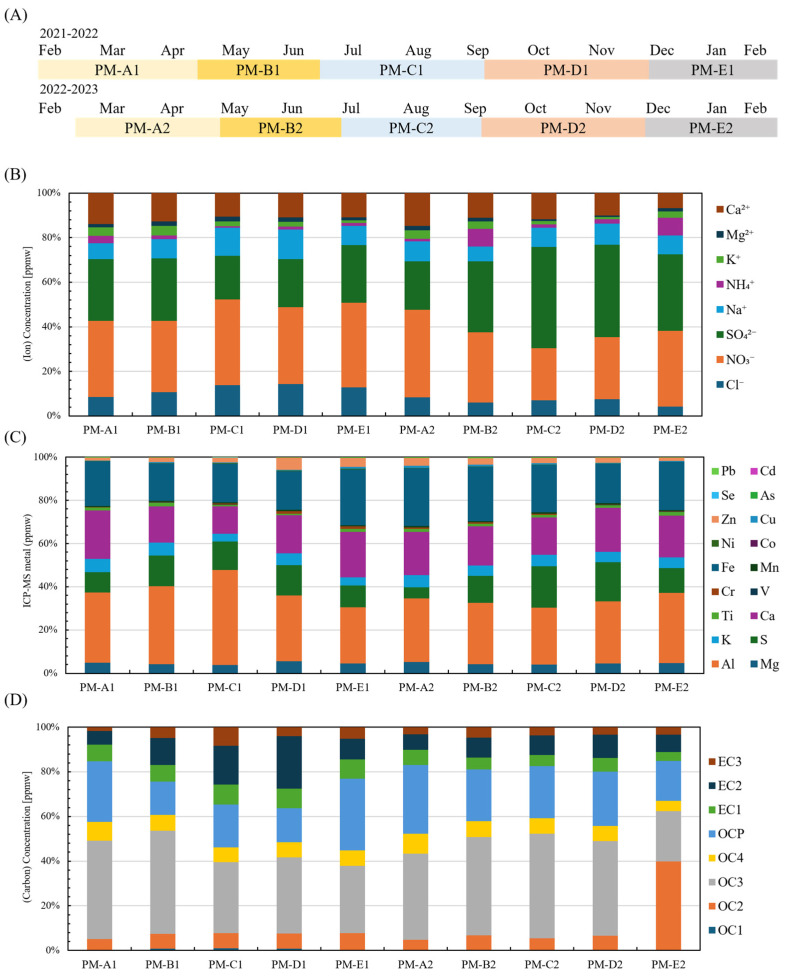
**PM_2.5_ collected during the summer (PM-C) showed elevated sulfur, reduced calcium, and decreased OCP levels**. (**A**) Sampling periods for PM_2.5_ collected from February 2021 to February 2023, divided into five seasonal periods per year (PM-A to PM-E). The numeral 1 after A to E indicates PM collected in 2021−2022 (PM-A1 to PM-E1), and 2 indicates PM collected in 2022−2023 (PM-A2 to PM-E2). (**B**) Relative proportions of major water-soluble ions (Cl^−^, NO_3_^−^, SO_4_^2−^, Na^+^, NH_4_^+^, K^+^, Mg^2+^, Ca^2+^) in each PM_2.5_ sample. (**C**) Elemental composition of PM_2.5_ samples, including crustal elements and heavy metals. (**D**) Carbonaceous fractions of PM_2.5_ determined by thermal–optical analysis, showing the distribution of organic carbon (OC1–OC4) and elemental carbon (EC1–EC3).

As shown in Figure 1A, PM_2.5_ was collected between February 2021 and February 2023 and classified into five seasonal periods (PM-A to PM-E), enabling inter-seasonal comparisons of particle composition. The proportions of major ionic components (Cl^−^, NO_3_^−^, SO_4_^2−^, Na^+^, NH_4_^+^, K^+^, Mg^2+^, Ca^2+^) are summarized in Figure 1B. Among these, Ca^2+^ showed the highest relative abundance in PM-A1 (13.9%) and PM-A2 (14.7%), while SO_4_^2−^ and NO_3_^−^ consistently dominated the anionic fraction, together accounting for more than 60% across all periods. Crustal elements, including Fe (>17%) and Al (>26%), were also present at high proportions in all samples (Figure 1C).

Clear seasonal variation was observed in several components. During the summer period (PM-C), Ca concentrations tended to be lower (12.7% and 17.3%), whereas S concentrations were markedly higher (13.3% and 19.1%). Although Fe concentrations were generally higher in winter (PM-E) than in summer (PM-C) across both years, Al did not show a consistent seasonal pattern (Pearson correlation coefficient r = −0.58). Given the limited number of seasonal composite samples, seasonal trends were evaluated using descriptive statistics and exploratory correlation analysis.

Figure 1D shows the distribution of carbonaceous fractions determined by thermal–optical analysis. Organic carbon (OC, >65.3%) accounted for a larger proportion than elemental carbon (EC, <34.7%), with OC3 being the most abundant fraction across seasons (22.5–47%). Pyrolyzed organic carbon (OCP) exhibited a modest increase during autumn and winter.

### 3.2. Intratracheal Administration of PM_2.5_ to Mice Increased BAL Cell Numbers and ROS Production

**Figure 2:**
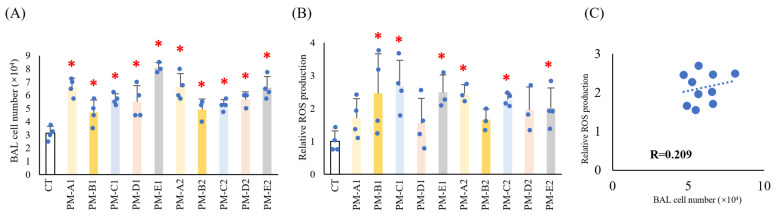
**Intratracheal administration of PM_2.5_ to mice increased BAL cell numbers and ROS production.** PM_2.5_ was collected from February 2021 to February 2023 as indicated. BALB/c mice were intratracheally administered with PBS (CT) or PM_2.5_ (100 μg/100 μL/mouse, *n* = 3–4) and dissected 24 h after administration. (**A**,**B**) BALF was collected, and BAL cell numbers were counted (**A**) and ROS production was measured using a DCFH-DA ROS Assay Kit (**B**). (**C**) The correlation between BAL cell numbers and ROS production, along with the correlation coefficient, was analyzed. The CT group (PBS, control) was set as 1.0. * *p* < 0.05 compared with CT.

To evaluate the biological effects of PM_2.5_, each seasonal sample was administered intratracheally to mice, and BAL cell counts and ROS production were quantified. Although some variability was observed across sampling periods, PM_2.5_ exposure consistently increased BAL cell numbers throughout the entire two-year collection period (Figure 2A).

ROS production was also induced by all PM_2.5_ samples, with particularly notable increases during PM-C and PM-E in both years (Figure 2B; PM-C1 and PM-C2, PM-E1 and PM-E2).

Correlation analysis revealed a weak (r = 0.21) but positive association between BAL cell numbers and ROS production (Figure 2C). Inclusion of the PBS-treated control group (CT) further strengthened this correlation (Supplementary Figure S2A), indicating that ROS generation is proportional to the magnitude of cellular infiltration induced by seasonal PM_2.5_

### 3.3. PM_2.5_ Administration Increased the Population of Neutrophil in BAL Cells

**Figure 3:**
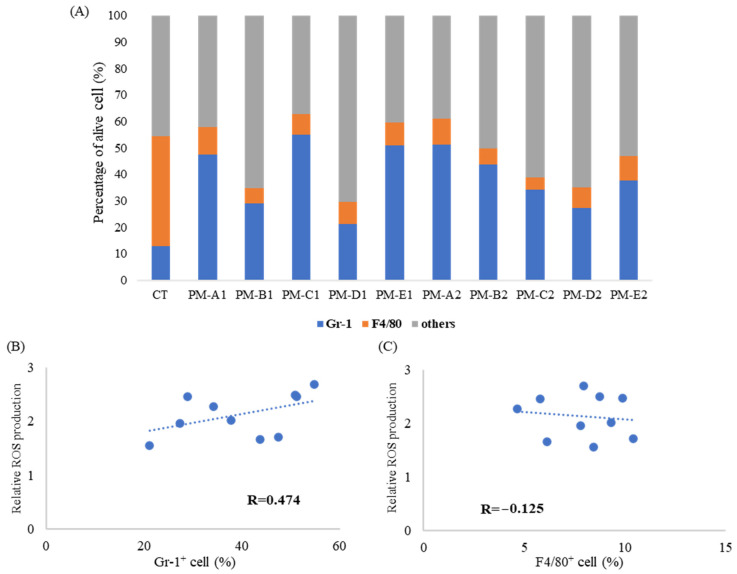
**PM_2.5_ administration increased the population of neutrophil in BAL cells.** PBS (CT) or PM shown in Figure 1A (100 μg/100 μL/mouse, *n* = 3–4) was administered intratracheally to BALB/c mice, and the mice were sacrificed 24 h after administration. (**A**) BAL cells were stained with each antigen-specific antibody and then analyzed by flow cytometry. The results show the proportions of Gr-1^+^ cells, F4/80^+^ cells, and remaining viable cells. (**B**,**C**) Correlations between Gr-1^+^ cells (**B**) or F4/80^+^ cells (**C**) and ROS production were analyzed. R represents the correlation coefficient.

As shown in Figure 3A, intratracheal administration of seasonal PM_2.5_ increased the total BAL cell numbers and markedly altered the composition of immune cell populations in the lung. Flow cytometric analysis demonstrated a clear increase in Gr-1^+^ neutrophils following PM_2.5_ exposure, accompanied by a relative decrease in F4/80^+^ macrophages compared with PBS-treated controls. These findings indicate that PM_2.5_ induces a shift toward neutrophil-dominant infiltration in the airways. The remaining cells, categorized as “other cells,” are expected to include lymphocytes and alveolar macrophages, with minor contributions from eosinophils and monocytes.

Correlation analysis (r = 0.47) further revealed that the proportion of Gr-1^+^ neutrophils was positively associated with ROS production (Figure 3B), suggesting that neutrophils are a major contributor to PM-induced oxidative activity. In contrast, the proportion of F4/80^+^ macrophages showed no correlation (r = −0.13) with ROS (Figure 3C), consistent with the observation that macrophage abundance decreases following PM exposure.

When the PBS control group (CT) was included, the positive correlation between neutrophil proportion and ROS production became even stronger (Supplementary Figure S2B). Under alternative analytical conditions, however, the correlation between neutrophils and ROS was reversed (Supplementary Figure S2C), highlighting the sensitivity of correlation patterns to data normalization and analytical parameters.

### 3.4. PM_2.5_ Administration Induced Inflammatory Cytokine Production

**Figure 4:**
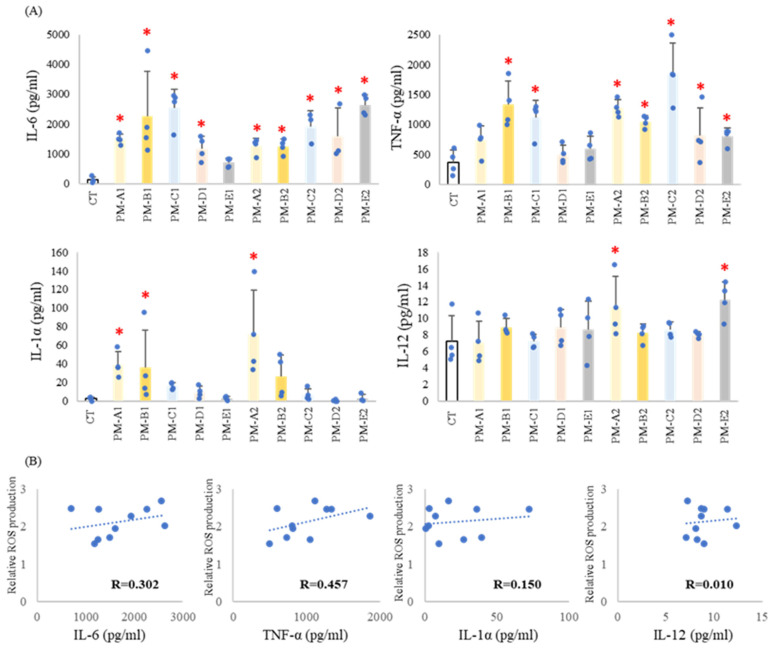
**PM_2.5_ administration induced inflammatory cytokine production.** PBS (CT) or PM_2.5_ (100 μg/100 μL/mouse, *n* = 3–4) were intratracheally administered to BALB/c mice, and mice were sacrificed 24 h after administration. BALF was collected, and cytokine levels were measured by ELISA. (**A**) The results showed IL-6, TNF-α, IL-1α, and IL-12 levels in BALF. CT, control group; * *p* < 0.05 compared with PBS. (**B**) The correlation between the mean cytokine concentration and ROS production in each group was analyzed. R represents the correlation coefficient.

To assess the inflammatory responses triggered by seasonal PM_2.5_, cytokine levels in BALF were quantified by ELISA. IL-6 and TNF-α levels were elevated following nearly all PM_2.5_ exposures, although the degree of induction varied across seasonal samples (Figure 4A). In contrast, IL-1α levels increased exclusively in response to PM-A samples in both years, indicating a season-specific pattern of IL-1α release. IL-12 levels remained largely unchanged regardless of PM_2.5_ exposure.

Correlation analysis revealed positive associations between ROS production and the levels of IL-6 (r = 0.30) and TNF-α (r = 0.46), supporting the concept that oxidative stress drives downstream pro-inflammatory cytokine production. Conversely, IL-1α and IL-12 showed no meaningful correlation with ROS (r = 0.15 and 0.01, respectively), suggesting that their regulation is governed by distinct upstream mechanisms independent of oxidative activity (Figure 4B).

Collectively, these findings indicate that most cytokine responses to PM_2.5_ align with patterns of ROS induction, while IL-1α behaves uniquely, showing both seasonal specificity and limited dependence on oxidative signaling pathways.

### 3.5. Mineral and Carbonaceous PM_2.5_ Components Drive Distinct Cellular Responses

Correlation analysis revealed that specific chemical constituents of PM_2.5_ were closely associated with distinct biological outcomes (Table 1). BAL cell counts showed a moderate positive correlation with nitrate (NO_3_^−^; r = 0.55) and a strong positive correlation with PAHs (r = 0.72), indicating that nitrate-rich and combustion-derived particles promote cellular infiltration into the airways.

ROS production displayed a moderate inverse correlation with ammonium (NH_4_^+^; r = –0.41) and a moderate positive correlation with BAL cell counts (r = 0.21), suggesting that reductions in secondary inorganic aerosol components coincide with increases in PM-induced oxidative activity.

Cytokine responses varied substantially by chemical species. IL-6 and IL-12 lacked strong correlations with any major constituent, implying that their induction reflects integrative inflammatory signaling rather than dependence on a single chemical driver. In contrast, TNF-α exhibited a strong negative correlation with PAHs (r = –0.62), suggesting that PAH-rich particles may suppress TNF-α production in this model.

Notably, IL-1α showed the most distinct pattern among cytokines: it strongly correlated with multiple mineral ions, including K^+^ (r = 0.80), Ca^2+^ (r = 0.81), and Mg^2+^ (r = 0.51). These findings indicate that mineral-rich particles—typically enriched in primary, crustal, and combustion-derived PM—serve as potent inducers of IL-1α release, distinguishing them from secondary inorganic components such as sulfate and ammonium.

### 3.6. Seasonal Peaks in PAHs Are Reproducible Across Years, Whereas Endotoxin Levels Fluctuate Substantially

Analysis of seasonal PAH concentrations revealed a highly reproducible pattern across the two-year sampling period (Table 2). In both years, PAH levels were consistently highest during period E, with moderately elevated concentrations during period A. Periods B and C repeatedly showed lower PAH abundance, demonstrating stable seasonal differences in the contribution of combustion-derived PM_2.5_ components.

In contrast, endotoxin levels displayed pronounced interannual variability. While seasons such as A and D showed comparable endotoxin levels between the two years, periods B and C differed markedly, with substantially higher endotoxin abundance observed in the second year. These findings indicate that biologically derived constituents of PM_2.5_, such as endotoxin, are more sensitive to year-to-year environmental fluctuations than chemically derived species like PAHs.

Together, the reproducible PAH pattern and the variable endotoxin levels highlight the differing environmental drivers of combustion-related versus biologically derived PM components.

### 3.7. PAH-Rich PM_2.5_ Enhances Cellular Infiltration While Endotoxin Drives Oxidative and Pro-Inflammatory Responses

Correlation analyses demonstrated that PAH-rich PM_2.5_ was strongly associated with enhanced cellular infiltration into the airways. BAL cell counts showed a robust positive correlation with PAH concentrations (r = 0.72), indicating that combustion-derived particles are potent drivers of inflammatory cell recruitment. In contrast, endotoxin levels exhibited a moderate negative correlation with BAL cell counts (r = –0.45), suggesting that endotoxin-rich PM_2.5_ does not promote cellular infiltration to the same extent as PAH-rich samples (Table 3).

ROS production showed a moderate negative correlation with endotoxin levels (r = –0.50), consistent with the idea that endotoxin-containing PM may trigger oxidative responses through pathways independent of cellular infiltration. Pro-inflammatory cytokines, including IL-6 and IL-12, exhibited only weak negative correlations with both PAHs and endotoxin, suggesting that their induction reflects integrated inflammatory signaling rather than dependence on specific PM constituents.

TNF-α displayed a strong negative correlation with PAHs (r = –0.62), supporting the hypothesis that PAH-rich particles suppress TNF-α production, a trend consistent with earlier analytical findings. IL-1α exhibited a moderate negative correlation with PAHs (r = –0.50) and no meaningful association with endotoxin, indicating that its regulation is driven predominantly by mineral-rich primary particles rather than combustion-related or biologically derived components.

Overall, these results highlight divergent roles of PAHs and endotoxin in shaping pulmonary responses: PAHs primarily enhance cellular infiltration, whereas endotoxin influences oxidative and select cytokine pathways through distinct mechanisms.

### 3.8. BAL Cell Counts and ROS Production Induced by PM_2.5_ Administration in TLR4 KO Mice Were Lower than Those in WT Mice

Because PM-C2 induced the highest TNF-α production among all PM_2.5_ samples, this sample was selected for downstream mechanistic analyses. PM-C2 was intratracheally administered to BALB/c (WT), TLR2 KO, and TLR4 KO mice to evaluate the involvement of TLR signaling pathways in PM-induced pulmonary inflammation.

**Figure 5:**
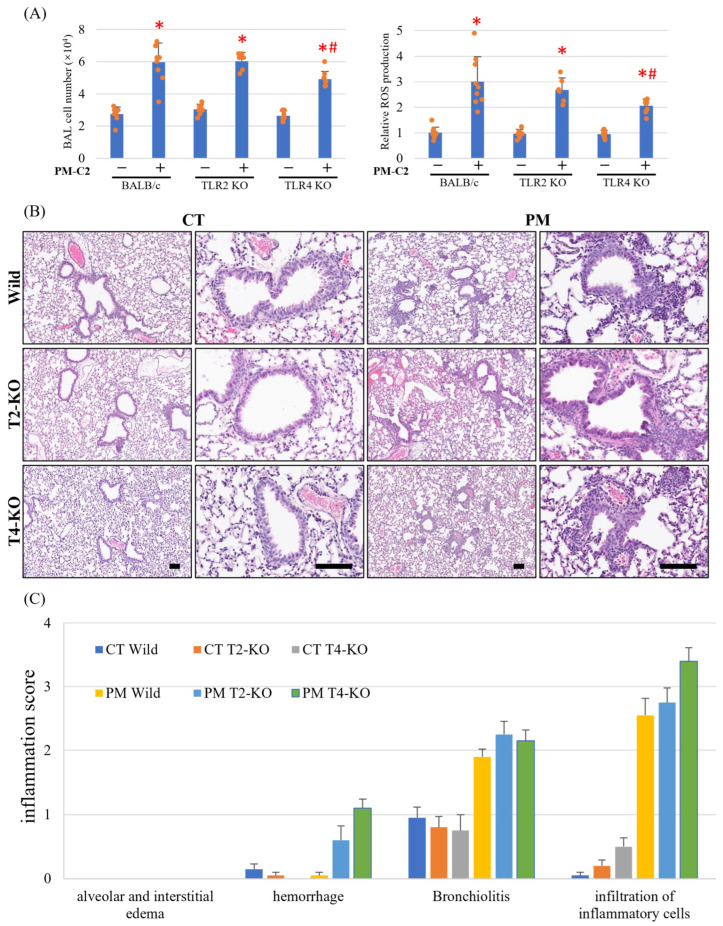
**BAL cell counts and ROS production induced by PM_2.5_ administration in TLR4 KO mice were lower than those in WT mice.** PBS (CT) or PM-C2 (100 μg/100 μL per mouse) was intratracheally administered to BALB/c, TLR2 KO, and TLR4 KO mice, and the mice were dissected 24 h after administration. (**A**) BALF was collected, BAL cell numbers were counted, and ROS production was measured using the DCFH-DA ROS Assay Kit. The results showed the relative fluorescence intensity of ROS production. (**B**,**C**) Lung samples were fixed and stained with hematoxylin and eosin. Representative photo is shown including black scale bar (200 μm). The severity of inflammation was scored in 10 fields of view, averaged, and summarized as a bar graph with error bars. Pathological images and graphs presented here are from a representative mouse in each experimental group (**C**). * *p* < 0.05 compared with CT of each mouse strain; # *p* < 0.05 compared with PM-C2-administered WT. WT;BALB/c mouse.

PM-C2 increased BAL cell counts and ROS production in all mouse strains; however, both responses were markedly attenuated in TLR4 KO mice compared with WT controls (Figure 5A). This partial reduction indicates that TLR4 contributes substantially, but not exclusively, to PM-induced cellular infiltration and oxidative activation.

Histopathological examination revealed PM-induced inflammatory changes throughout multiple lung regions, including bronchioles and alveolar spaces (Figure 5B). Interestingly, despite showing reduced BAL cell counts and ROS production, TLR4 KO mice exhibited higher inflammation scores—specifically increased hemorrhage, bronchiolitis, and inflammatory cell accumulation—relative to WT mice (Figure 5C).

These findings suggest a dual role for TLR4: while it facilitates PM-induced inflammatory activation, it may also contribute to resolution or containment of lung injury. Loss of TLR4 thus reduces acute cellular and oxidative responses but predisposes the lung to dysregulated or prolonged inflammation following PM_2.5_ exposure.

### 3.9. IL-6 and TNF-α Levels in TLR4 KO Mice Were Lower than Those in WT Mice

**Figure 6:**
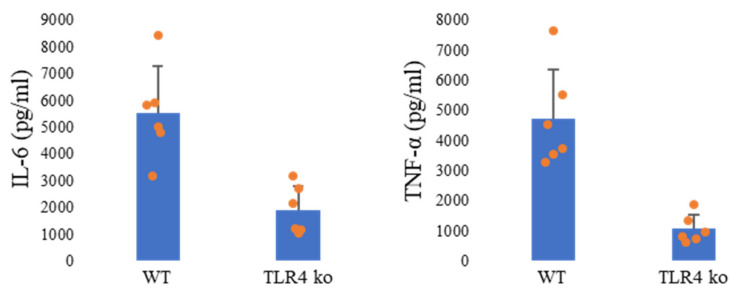
**IL-6 and TNF-α levels in TLR4 KO mice were lower than those in WT mice.** PBS (CT) or PM-C2 (100 μg/100 μL/mouse, *n* = 6) was intratracheally administered to BALB/c or TLR4 KO mice, and the mice were dissected 24 h after administration. BALF was collected, and IL-6 and TNF-α levels were measured by ELISA. WT;BALB/c mouse.

To further examine the contribution of the TLR4 signaling pathway to PM_2.5_-induced inflammation, IL-6 and TNF-α levels were quantified in the BALF of WT and TLR4 KO mice following PM-C2 administration. Both cytokines were markedly reduced in TLR4 KO mice compared with WT controls (Figure 6), reinforcing the conclusion that TLR4 plays a key role in promoting pro-inflammatory cytokine responses to PM_2.5_.

These findings, together with the reduced BAL cell infiltration and ROS production observed in TLR4 KO mice, demonstrate that TLR4 regulates multiple facets of the pulmonary inflammatory response. However, the heightened histopathological inflammation observed in TLR4 KO mice (Section 3.8) suggests that TLR4 also contributes to injury resolution, indicating a dual and context-dependent function during PM-induced lung inflammation.

## 4. Discussion

Airborne PM has emerged as a significant environmental issue because of its adverse effects on human health, which fluctuate with seasonal changes and human activities. Although monitoring efforts commonly emphasize PM concentrations, the chemical makeup of these particles is equally critical [38].

In this study, we systematically analyzed PM_2.5_ collected over two consecutive years from a single urban site in Japan and demonstrated that both its chemical composition and biological effects varied markedly by season. Seasonal fluctuations in PM_2.5_ characteristics, including oxidative potential and toxicity, have been widely reported in previous studies [12,13]. Consistent with these reports, we observed clear seasonal patterns in the present work, including elevated sulfur levels in summer and increased PAH concentrations in winter [39]. Importantly, these seasonal trends are consistent with observations reported in other regions outside Japan. Studies conducted in Europe, North America, and East Asia have similarly shown higher contributions of secondary sulfate in summer and increased levels of PAHs and combustion-related components during winter, reflecting enhanced photochemical activity in warmer seasons and the accumulation of combustion emissions under stagnant meteorological conditions in colder months [40]. These parallels suggest that the seasonal variability observed in the present study reflects common atmospheric processes rather than region-specific phenomena. These differences likely reflect seasonal variations in secondary aerosol formation and the accumulation of combustion-derived pollutants.

It is well investigated that PM_2.5_ administration induces systemic inflammation and ROS production [41]. Biologically, PM_2.5_ collected in summer (PM-C) and winter (PM-E) induced the highest levels of ROS production and BAL cell infiltration. Combustion-derived components such as PAHs and elemental carbon (EC) possess strong intrinsic redox activity, which contributes to ROS generation and oxidative stress in lung tissue [26,27]. The correlation analysis further suggested that multiple chemical constituents, including nitrates, vanadium, iron, and OCP fractions, are involved in shaping PM_2.5_-induced inflammatory responses. Notably, the direction and magnitude of these correlations were sensitive to the normalization strategy applied, as shown in Supplementary Figure S2. This analytical sensitivity underscores the need for cautious interpretation of correlation strength and direction and supports the use of these analyses primarily as exploratory and hypothesis-generating rather than confirmatory. Although individual components such as PAHs and mineral constituents were analyzed separately in this study, interactive or synergistic effects among PM_2.5_ components may further modulate toxicity and warrant investigation in future studies. Mechanistically, several of the identified constituents have been implicated in PM_2.5_-induced inflammatory responses in previous studies. Transition metals such as iron and vanadium can catalyze redox reactions, leading to excessive ROS generation and activation of oxidative stress-responsive signaling pathways. PAHs and carbonaceous fractions, including EC and organic carbon, are known to induce epithelial injury and macrophage activation through both oxidative and receptor-mediated mechanisms, thereby promoting the release of pro-inflammatory cytokines and chemokines. These findings underscore the importance of particle composition, rather than mass concentration alone, in determining the health effects of ambient PM_2.5_. While PM_2.5_ mass is commonly used as a regulatory metric, our results support the growing body of evidence that specific chemical constituents and their combinations play a decisive role in driving biological responses. Such compositional insights may contribute to more refined risk assessment strategies and targeted mitigation policies aimed at reducing the most toxic components of ambient particulate matter.

A strong association was observed between ROS levels and the proportion of neutrophils in BAL cells. Neutrophils are known to be major sources of ROS in PM-induced pulmonary inflammation [21], and excessive ROS can activate redox-sensitive transcription factors such as NF-κB, leading to the production of pro-inflammatory cytokines including IL-6 and TNF-α [28,42]. In line with these mechanisms, PM-C2, one of the samples with the strongest ROS-inducing capacity, elicited the highest levels of TNF-α. In addition, PAHs strongly correlated with BAL cell counts in the present study. PAHs are known to activate the aryl hydrocarbon receptor (AhR) and can be enzymatically converted into redox-active quinone intermediates, thereby amplifying oxidative stress and inflammatory signaling [7,43]. Our results support the notion that PM_2.5_-induced oxidative stress and neutrophil-driven inflammation are central mechanisms contributing to pulmonary injury, highlighting the importance of chemical composition in shaping these biological responses. It should be noted that ambient PM_2.5_ represents a complex mixture of multiple PAH species rather than isolated compounds, and human exposure occurs predominantly under such mixed conditions. While certain PAHs classified as Group B2 by the U.S. EPA exhibit higher toxic potency at the individual compound level, the present study was designed to evaluate the integrated biological effects of environmentally relevant PAH mixtures within seasonal PM_2.5_ samples. Our findings therefore reflect the combined influence of co-existing PAHs and other particle-associated components, which may interact to modulate oxidative stress and inflammatory responses. This mixture-based perspective is particularly relevant for assessing real-world health effects of ambient particulate matter.

Although intracellular ROS production was assessed using DCFH-DA, a widely applied probe for oxidative stress, this method does not distinguish between specific ROSs and their cellular sources. Thus, the observed signals should be interpreted as an index of overall oxidative burden rather than individual ROS. Given that PM_2.5_ components such as transition metals and organic compounds can generate distinct ROS through different mechanisms, future studies employing more specific probes or complementary approaches will be necessary to delineate the precise ROS and cellular origins involved.

Previous studies have identified TLR4 as a key receptor mediating the recognition of environmental particles and subsequent pro-inflammatory signaling, primarily through the activation of NF-κB and ROS-generating pathways [42,44]. To further investigate the involvement of innate immune signaling pathways in PM-driven inflammation, we compared the effects of PM_2.5_ exposure in wild-type, TLR2-deficient, and TLR4-deficient mice. TLR4 is known to recognize pathogen-associated molecules such as LPS, as well as environmental pollutants including PAHs and certain metals [36,42]. In our study, TLR4 knockout mice exhibited significantly lower BAL cell counts, ROS production, and cytokine levels than wild-type mice, supporting the idea that TLR4 partially mediates the inflammatory response to PM_2.5_.

Interestingly, histopathological analysis revealed more severe inflammatory changes in TLR4-deficient mice compared with wild-type animals, despite reduced BAL cellularity and cytokine levels. This apparent paradox is consistent with previous studies demonstrating dual roles for TLR4, not only as a pro-inflammatory receptor but also as a critical regulator of inflammation resolution and tissue protection [45,46]. In the absence of TLR4 signaling, impaired resolution processes—such as defective clearance of damaged cells, delayed removal of inhaled particles, or dysregulated termination of inflammatory signaling—may contribute to persistent tissue injury [47]. In addition, TLR4 has been implicated in maintaining epithelial barrier integrity and coordinating appropriate cell death pathways, including apoptosis versus necrotic or lytic forms of cell death. Disruption of these processes could exacerbate local tissue damage and histopathological inflammation, even in the context of attenuated cytokine production.

Collectively, these findings suggest that TLR4 plays a dual role in pulmonary responses to PM_2.5_, contributing both to the initiation of inflammatory signaling and to its subsequent resolution. Consequently, TLR4 deficiency may predispose the lung to dysregulated or non-resolving inflammation, resulting in enhanced histopathological injury despite reduced conventional inflammatory readouts.

While the chemical composition of PM_2.5_ is known to vary spatially depending on emission sources and meteorological conditions, the present study analyzed PM_2.5_ samples collected from a single urban area. Consequently, the biological responses observed here may differ in regions with distinct particle compositions, and caution should be exercised when extrapolating these findings to other geographical settings.

Although intratracheal instillation offers a well-controlled and reproducible exposure paradigm, it does not fully recapitulate real-life inhalation exposure. In particular, differences in particle deposition patterns, clearance kinetics, and the temporal dynamics of inflammatory responses should be considered when extrapolating these findings to environmental conditions. Accordingly, the seasonal differences observed in this study are not directly predictive of health effects associated with airborne inhalation exposure and should be interpreted in the context of these limitations.

One of the key findings of this study is the strong positive correlation between IL-1α—an alarmin derived from epithelial cells and macrophages that induces particulate matter-induced pulmonary inflammation—and several mineral components, such as K, Ca, and Mg. These species are enriched in combustion-derived primary particles and are typically more abundant in winter PM_2.5_, reflecting biomass burning, soil resuspension, and mixed combustion sources. In fact, PM_2.5_ induced the highest levels of IL-1α in winter. The preferential association of IL-1α with these components suggests that mineral-rich primary particles may be particularly potent in triggering IL-1α-mediated alarmin responses. This interpretation is consistent with mechanistic studies demonstrating that micro- and nanoparticles, including silica, can induce alveolar macrophage death and subsequent IL-1α release, thereby initiating acute lung inflammation [48,49]. Human exposure studies have similarly shown that inhalation of organic dust elevates BALF IL-1α [50], further supporting the notion that particle composition critically determines IL-1α-dependent pathways.

In contrast, IL-1α showed inverse correlations with secondary inorganic aerosol components such as sulfate and sulfur, which are more abundant in summer and represent photochemically generated secondary particles. This negative association suggests that secondary inorganic aerosols have comparatively weak capacity to induce IL-1α release, despite contributing substantially to PM_2.5_ mass in warm seasons. Such seasonal divergence in IL-1α responsiveness is consistent with prior observations that winter PM_2.5_ exhibits stronger oxidative and inflammatory potential than summer PM_2.5_, largely due to differences in carbonaceous and metal constituents [51].

IL-1α is produced by multiple lung cell types, most notably epithelial cells and alveolar macrophages, each contributing differently to particle-induced lung injury. Epithelial-derived IL-1α primarily functions as an alarmin, being rapidly released upon cellular stress or damage, whereas macrophage-derived IL-1α reflects active inflammatory signaling following particle phagocytosis. The distinct seasonal behavior of IL-1α observed in this study may therefore reflect differences in the relative contribution of these cellular sources, potentially driven by seasonal variability in PM_2.5_ composition and toxicity. Although the present study does not directly resolve the cellular origin of IL-1α, this distinction represents an important mechanistic consideration and warrants further investigation in future studies.

The identification of IL-1α as a sensitive and seasonally responsive marker of PM_2.5_-induced lung inflammation may have important translational implications for environmental health research. As an alarmin predominantly released upon epithelial injury, IL-1α could serve as an early sentinel indicator reflecting the inflammatory potential of particulate matter before overt immune cell recruitment becomes evident. From a human exposure perspective, seasonal variation in IL-1α responses may aid in risk stratification by highlighting periods during which specific PM_2.5_ compositions exert heightened biological activity. Furthermore, IL-1α-centered responses may provide a mechanistic bridge between particle composition and downstream inflammatory outcomes, supporting its potential utility as a biomarker in exposure assessment studies. Future investigations integrating human biomonitoring data, such as airway or circulating IL-1α levels, together with compositional analyses of ambient PM, would help clarify its applicability for evaluating population-level susceptibility and seasonal health risks associated with air pollution.

Taken together, these findings support a model in which IL-1α functions as an early sentinel of lung injury preferentially activated by combustion-related, mineral-rich primary particles, whereas secondary inorganic aerosols exert modest effects on this alarmin pathway. Because IL-1α not only signals tissue injury but also amplifies downstream inflammatory cascades, the enhanced activation of IL-1α by winter PM_2.5_ may represent a key mechanism linking seasonal variations in PM composition to the worsening of respiratory symptoms. These results highlight the importance of particle composition—beyond mass concentration alone—in determining the health effects of ambient PM_2.5_ and underscore IL-1α as a sensitive biomarker for evaluating the toxicity of primary combustion particles.

## 5. Conclusions

Taken together, our findings provide new insight into how seasonal shifts in PM_2.5_ composition shape oxidative stress, neutrophil-mediated inflammation, and innate immune signaling in the lungs. By integrating two full years of data on chemically characterized PM_2.5_ with in vivo functional analyses, this study highlights the importance of particle composition, rather than mass concentration alone, in determining biological toxicity.

PAH-rich particles, which consistently peak in winter, strongly promoted cellular infiltration, whereas mineral-rich primary particles showed a distinct capacity to induce IL-1α, an early alarmin that amplifies downstream inflammatory cascades. In contrast, secondary inorganic aerosols contributed minimally to IL-1α-driven pathways, underscoring composition-specific biological effects among seasonal PM_2.5_ species.

Mechanistically, ROS generation tightly correlated with neutrophil recruitment and cytokine production, positioning oxidative stress as a key upstream driver of PM-induced inflammation. Experiments using TLR-deficient mice further demonstrated that TLR4 partially mediates PM-induced cellular and cytokine responses, while simultaneously influencing the resolution of tissue injury, revealing a dual, context-dependent role for this receptor.

Overall, this work emphasizes the need for the consideration of seasonal and compositional variability in regulatory and health-risk frameworks for PM_2.5_. Such mechanistic insights may also inform the development of targeted interventions that mitigate PM-induced oxidative lung injury, particularly during high-risk winter pollution events.

Importantly, although this study was conducted using PM_2.5_ collected at a single urban site in Japan, the observed relationships between seasonal particle composition and inflammatory responses are consistent with atmospheric processes and emission patterns reported globally. Similar seasonal enrichment of combustion-derived PAHs in winter and secondary aerosols in summer has been documented in many regions worldwide, suggesting that the composition-dependent biological effects identified here may be broadly applicable to other urban and industrialized areas. In addition, previously established in vitro experimental systems using differentiated neutrophils [52] provide a complementary platform for dissecting the underlying molecular mechanisms, including the contribution of TLR4 signaling. Application of these systems to seasonal PM_2.5_ samples represents an important and logical extension of the present findings.

## Figures and Tables

**Table 1:** **Pearson correlation matrix between PM_2.5_ chemical components and pulmonary oxidative and inflammatory responses.**

	Cl^−^	NO_3_^−^	SO_4_^2−^	Na^+^	NH_4_^+^	K^+^	Mg^2+^	Ca^2+^	BAL CELL		
**BAL CELL**	**0.11**	**0.55**	−**0.25**	−**0.13**	−**0.09**	−**0.23**	−**0.12**	−**0.04**		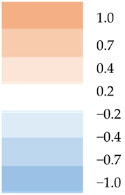	
**ROS**	**0.17**	−**0.08**	−**0.05**	**0.20**	−**0.41**	**0.23**	**0.25**	**0.25**	**0.21**		
**IL-6**	−**0.23**	−**0.16**	**0.16**	**0.17**	**0.16**	**0.17**	**0.09**	−**0.39**	−**0.40**		
**TNF-α**	−**0.30**	−**0.48**	**0.41**	−**0.19**	−**0.17**	**0.15**	−**0.13**	**0.31**	−**0.48**		
**IL-1α**	−**0.06**	**0.40**	−**0.45**	−**0.25**	−**0.12**	**0.80**	**0.51**	**0.81**	−**0.03**		
**IL-12**	−**0.43**	**0.19**	**0.02**	−**0.10**	**0.34**	**0.22**	**0.11**	−**0.28**	**0.22**		
	**Mg**	**Al**	**S**	**K**	**Ca**	**Ti**	**V**	**Cr**	**Mn**		
**BAL CELL**	**0.30**	−**0.27**	−**0.58**	−**0.29**	**0.58**	**0.04**	**0.65**	**0.32**	**0.29**		
**ROS**	−**0.57**	**0.53**	**0.19**	−**0.02**	−**0.71**	**0.14**	−**0.35**	−**0.19**	−**0.59**		
**IL-6**	−**0.45**	**0.70**	**0.30**	−**0.08**	−**0.56**	**0.26**	−**0.24**	−**0.62**	−**0.55**		
**TNF-α**	−**0.57**	**0.04**	**0.29**	**0.18**	−**0.41**	**0.26**	−**0.06**	−**0.29**	−**0.22**		
**IL-1α**	**0.24**	**0.12**	−**0.69**	**0.53**	**0.13**	**0.25**	**0.14**	−**0.15**	−**0.04**		
**IL-12**	**0.37**	−**0.22**	−**0.38**	**0.17**	**0.20**	**0.43**	**0.72**	−**0.07**	**0.32**		
	**Fe**	**Co**	**Ni**	**Cu**	**Zn**	**As**	**Se**	**Cd**	**Pb**		
**BAL CELL**	**0.53**	**0.57**	**0.16**	**0.21**	**0.15**	−**0.46**	−**0.39**	**0.04**	−**0.15**		
**ROS**	−**0.27**	−**0.36**	−**0.15**	**0.14**	−**0.24**	−**0.25**	−**0.07**	−**0.14**	−**0.04**		
**IL-6**	−**0.51**	−**0.60**	−**0.51**	−**0.59**	−**0.66**	**0.00**	−**0.14**	−**0.08**	−**0.47**		
**TNF-α**	**0.07**	−**0.40**	−**0.26**	**0.31**	−**0.34**	**0.09**	**0.13**	−**0.32**	**0.34**		
**IL-1α**	**0.33**	**0.15**	−**0.23**	**0.41**	−**0.04**	−**0.17**	−**0.64**	−**0.76**	**0.21**		
**IL-12**	**0.42**	**0.38**	−**0.14**	**0.14**	**0.08**	−**0.15**	−**0.19**	−**0.01**	**0.14**		
	**OC1**	**OC2**	**OC3**	**OC4**	**OCP**	**EC1**	**EC2**	**EC3**	**OC**	**EC**	
**BAL CELL**	−**0.34**	**0.21**	−**0.57**	**0.10**	**0.69**	**0.22**	−**0.33**	−**0.16**	**0.22**	−**0.22**	
**ROS**	**0.42**	−**0.20**	**0.27**	**0.05**	−**0.31**	**0.08**	**0.10**	**0.53**	−**0.21**	**0.21**	
**IL-6**	**0.49**	**0.51**	−**0.17**	−**0.53**	−**0.64**	−**0.32**	**0.05**	**0.29**	−**0.03**	**0.03**	
**TNF-α**	−**0.14**	−**0.21**	**0.54**	**0.17**	−**0.04**	−**0.38**	−**0.29**	**0.12**	**0.26**	−**0.26**	
**IL-1α**	−**0.12**	−**0.34**	**0.35**	**0.78**	**0.29**	**0.13**	−**0.30**	−**0.22**	**0.24**	−**0.24**	
**IL-12**	−**0.22**	**0.69**	−**0.48**	−**0.29**	−**0.04**	−**0.49**	−**0.24**	−**0.29**	**0.35**	−**0.35**	

Values represent Pearson correlation coefficients (r). Positive and negative correlations are indicated by warm and cool colors, respectively. Color shading indicates the strength and direction of correlations, with orange representing positive correlations and blue representing negative correlations. Darker shading corresponds to higher absolute correlation coefficients (0.7, 0.4, and 0.2). Abbreviations: bronchoalveolar lavage (BAL), reactive oxygen species (ROS), interleukin-6 (IL-6), tumor necrosis factor-α (TNF-α), interleukin-1α (IL-1α), interleukin-12 (IL-12), organic carbon (OCP), elemental carbon (EC), and pyrolyzed organic carbon (OCP).

**Table 2:** **PAH and endotoxin levels in PM_2.5_ samples collected across different seasons.**

A1.	PM-A1	PM-B1	PM-C1	PM-D1	PM-E1	PM-A2	PM-B2	PM-C2	PM-D2	PM-E2
PAHs	11.8	7.9	7.5	10.5	17.9	8.8	11.2	9.1	12.9	15.4
(ng/mg)										
Endotoxin	4.2	3.5	1.1	3.1	2.9	3.1	12.4	6.6	6.7	2.3
(EU/mg)										

Abbreviation: polycyclic aromatic hydrocarbons (PAHs).

**Table 3:** **Pearson correlation analysis between PM_2.5_ PAH and endotoxin levels with *inflammatory or oxidative stress indicators*.**

	PAHs	Endotoxin	
**BAL CELL**	**0.72**	−**0.45**	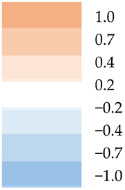
**ROS**	−**0.20**	−**0.50**	
**IL-6**	−**0.25**	−**0.28**	
**TNF-α**	−**0.62**	**0.22**	
**IL-1α**	−**0.50**	−**0.03**	
**IL-12**	**0.21**	−**0.24**	

Values represent Pearson correlation coefficients (r). Positive and negative correlations are indicated by warm and cool colors, respectively. Color shading indicates the strength and direction of correlations, with orange representing positive correlations and blue representing negative correlations. Darker shading corresponds to higher absolute correlation coefficients (0.7, 0.4, and 0.2). Abbreviations: polycyclic aromatic hydrocarbons (PAHs), bronchoalveolar lavage (BAL), reactive oxygen species (ROS), interleukin-6 (IL-6), tumor necrosis factor-α (TNF-α), interleukin-1α (IL-1α), and interleukin-12 (IL-12).

## Data Availability

The original contributions presented in this study are included in the article/Supplementary Materials. Further inquiries can be directed to the corresponding author.

## Supplementary Materials

The following supporting information can be downloaded at: https://www.mdpi.com/article/10.3390/antiox15010089/s1. Figure S1: Intratracheal administration of increased the population of Gr-1^+^ cell and decreased population of F4/80^+^ cell in BAL cell. Figure S2: Intratracheal administration of PM_2.5_ increases BAL cell and correlates with enhanced ROS production.
