# Supplementary material for: Seasonal Variation in PM2.5 Composition Modulates Oxidative Stress and Neutrophilic Inflammation with Involvement of TLR4 Signaling
Source: Antioxidants (Basel). 2026 Jan 9;15(1):89. doi: 10.3390/antiox15010089 (PMC12837680; doi:10.3390/antiox15010089)
Supplement: Supplementary file 1 [file antioxidants-15-00089-s001.zip › antioxidants-4062009-supplementary.pdf]

Figure S1.

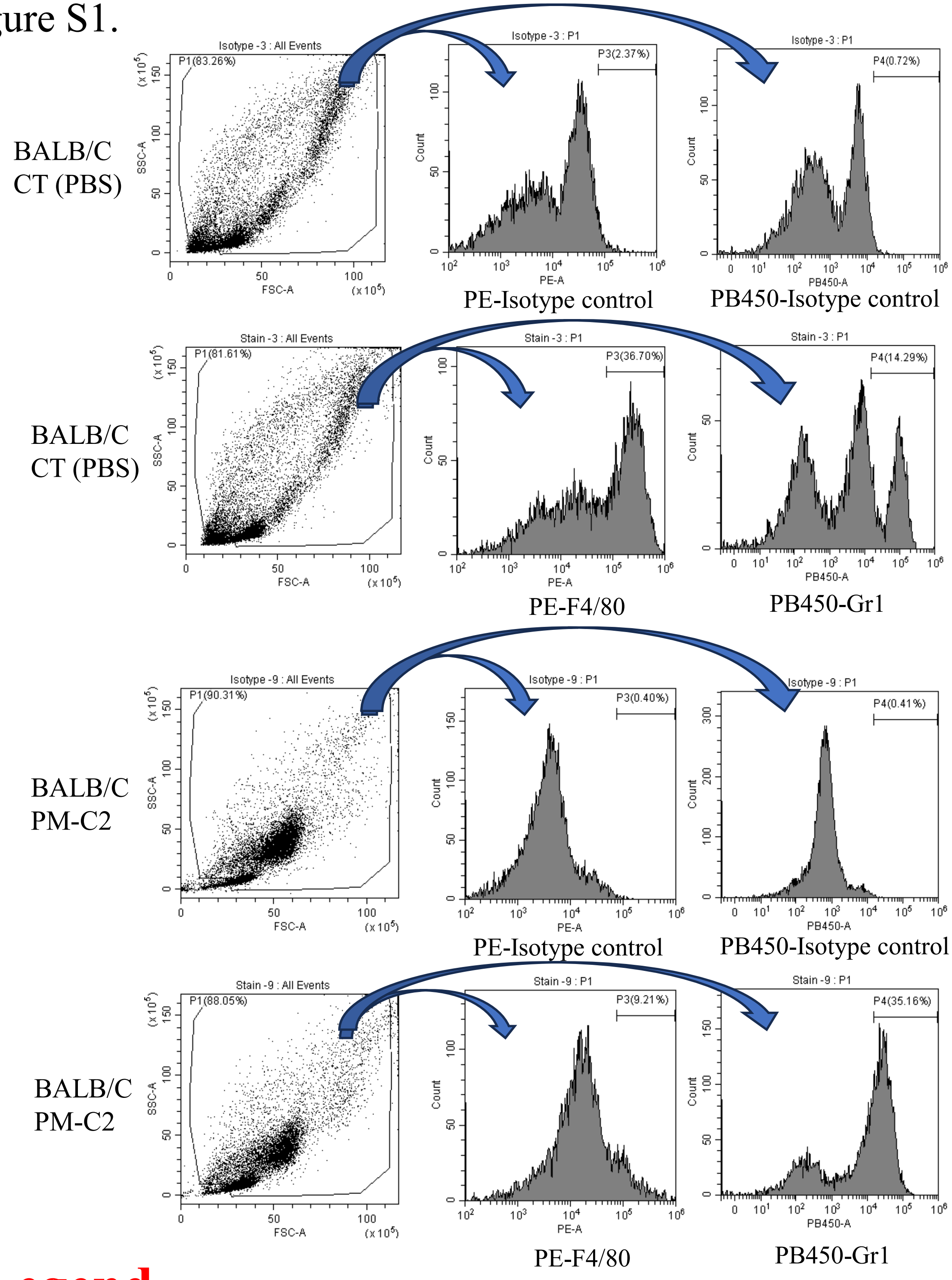

Legend

Intratracheal administration of increased the population of Gr-1^{+} cell and decreased population of F4/80^{+} cell in BAL cell.

PBS (CT) or PM shown in Figure (100 μg/100 μl/mouse) was administered intratracheally to BALB/c mice, and the mice were sacrificed 24 hours after administration. BAL cells were stained with each antigen-specific antibody or isotype control and then analyzed by flow cytometry. Gating strategy of flow cytometry was shown. The results show the proportions of Gr-1^{+} cells and F4/80^{+} cells in BAL cell.

Figure S2.

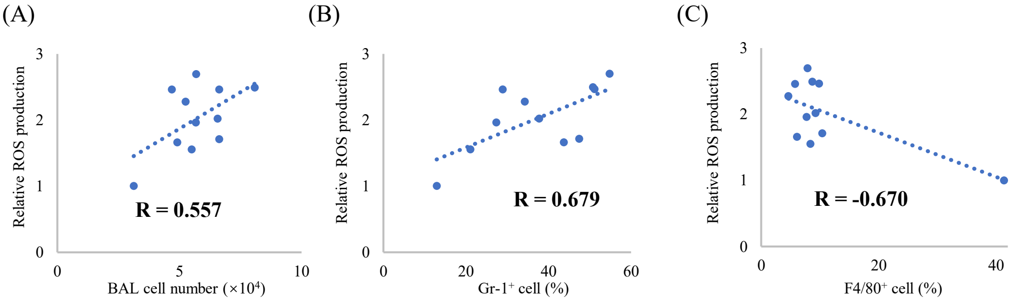

Legend

Intratracheal administration of PM2.5 increases BAL cell and correlates with enhanced ROS production.

PM2.5 was collected from 2021.2-2023.2 as indicated period. PBS (CT) or PM2.5(100μg/100ul/mouse, n=3 or 4) were intratracheal administered to BALB/c mice and mice were dissected 24 hours after the administration. BAL cell were stained with Percpcy5.5-CD11b antibody, Violet450-Gr1 antibody and PE-F4/80 antibody at 4 ^{o}C in 30 minuets. The correlation and correlation coefficient between BAL cell number(A), Gr-1^{+} cell (B) or F4/80^{+} cell (C) and ROS production was analyzed. Results showed that there is a negative correlation between BAL cell number and ROS production.

CT (PBS, control group) were served as 1.0. The correlation between BAL cell and ROS were showed. BALF, bronchoalveolar lavage fluid; ROS, reactive oxygen species; *p<0.05 compared with PBS
